# Anemia following zinc treatment for Wilson’s disease: a case report and literature review

**DOI:** 10.1186/s12876-019-1038-5

**Published:** 2019-07-09

**Authors:** Sha Cai, Jing-Yu Gong, Jing Yang, Jian-She Wang

**Affiliations:** 1 Department of Pediatrics, Jinshan Hospital of Fudan University, 1508 Longhang Road, Jinshan District, Shanghai, 201508 China; 2 Center for Pediatric Liver Diseases, Children’s Hospital of Fudan University, Shanghai, 201102 China

**Keywords:** Wilson’s disease, Hepatolenticular degeneration, Zinc, Hypocupremia, Anemia

## Abstract

**Background:**

Zinc therapy is considered an effective and safe treatment for Wilson’s disease. Hypocupremia-related anemia is rarely reported after long-term zinc administration or combination therapy with copper-chelating agent.

**Case presentation:**

We herein report a 12-year-old girl with pre-symptomatic Wilson’s disease diagnosed 5 years ago who presented with severe anemia after high-dose oral zinc for 4 years and 4 months. Her hemoglobin was gradually restored to the normal range after the adjustment of zinc dose and diet therapy for 4 months. A review of the literature revealed eight patients with hypocupremia-associated anemia following zinc therapy for Wilson’s disease, including 7 adults and 1 child. The only child patient was a 16-year-old boy, in whom the zinc therapy was succession to penicillamine administration.

**Conclusions:**

This is the first report worldwide that a child developed severe anemia following high-dose single zinc administration for Wilson’s disease. It highlights the importance of regular follow-up during zinc treatment and the involvement of specialists in the long-term management of Wilson’s disease. We hope that this will alert pediatricians the issue of zinc over-treatment.

## Background

Wilson’s disease (WD), first reported by Kinnear Wilson in 1912 [1], is an autosomal recessive inherited disease. It is caused by dysfunction of the ATP7B gene [2] which affects the formation of ceruloplasmin and impairs copper secretion into bile, resulting in excessive copper deposition in the liver, brain and other tissues, and leads to a series of clinical manifestations including hepatic, neurological and psychiatric symptoms [3–6].

Zinc is believed to be a safe and effective first-line drug in treatment of WD. Gastrointestinal reactions and hepatic deterioration are relative common side effects of zinc therapy [7–9]. Hypocupremia-associated anemia after long-term copper depletion therapy in WD is rare. Only eight patients with anemia after a long period of zinc therapy for WD have been reported [10–17]. Here, we report a 12-year-old girl with anemia following a relative short-term zinc treatment for WD.

## Case presentation

A 12-year-old girl was first noted to have elevated alanine aminotransferase (379 U/L, normal: 0–40 U/L) and aspartate aminotransferase (218 U/L, normal, 0–40 U/L) when abdominal pain occurred at the age of 6 years. Viral serological markers (hepatotropic viruses, Epstein-Barr virus, and cytomegalovirus) were negative. Renal function tests, electrolyte and coagulation function were unremarkable. At the age of 7-year-old, her transaminase levels were still abnormal, and further investigations revealed low serum ceruloplasmin (< 0.079 g/L, cut-off value: 0.2 g/L) and increased 24-h urinary copper excretion (360 μg, cut-off value: 40 μg) [18]. No corneal Kayser-Fleischer ring was observed by slit-lamp examination of her eyes. There was no family history of liver disease. Her parents and biological younger sister all had normal liver function tests.

ATP7B (NM_−_ 000053) sequencing identified two novel heterozygous mutations, c.2122-1G > T (paternal) and c.3044 T > C, p. (L1015P) (maternal). Neither of these mutations have been described and recorded in the Exome Aggregation Consortium Server (http://www.exac.broad-institute.org) and the Thousand Genomes Project (http://www.1000genomes.org). They were predicted to be disease-causing by MutationTaster (http://www.mutationtaster.org). L1015P was also predicted to be probably damaging with a score of 1.000 by PolyPhen2 (http://genetics.bwh.harvard.edu/pph2/index.shtml), and was predicted to affect protein function with a score of 0.00 by SIFT (http://sift.jcvi.org/www/SIFT_seq_submit2.html). The patient was treated with a low-copper diet, and zinc gluconate (150 mg of elemental zinc daily in three divided doses, at least half an hour before meals, which was gradually increased to 240 mg/day due to persistently elevated liver aminotransferases and 24-h urinary copper excretion). She complained of occasional abdominal pain during the first three months of zinc therapy. Liver function tests returned to normal after six months. She has continued to take zinc at a dose of 240 mg/day, and avoids foods with high copper content. Since her age of 8 years, no further visits to physicians were made until the patient was 11 years and 4 months old, when she presented with a pale face and fatigue. Whole blood cell counts disclosed severe normocytic anemia and neutropenia. She attended a pediatric hematology clinic and underwent anemia-related tests with no remarkable findings including negative direct and indirect Coombs tests, normal glucose-6-phosphate dehydrogenase activity and serum ferritin level. Serum zinc level was 530 μg/dL(normal:75–150 μg/dL) and serum copper level was 127 μg/L(normal: 800–1290 μg/L). Bone marrow cytology showed myelodysplastic syndrome. She received transfusion of 2 units of erythrocyte suspension and continued oral zinc at her previous dose. There was no significant improvement in hematological parameters.

She returned to us 6 months later. Physical examinations revealed a pale face, mild hepatomegaly and abnormal gait, no splenomegaly, no ascites, no personality change, no paresthesias, no myasthenia, no hypertonia or hyperreflexia. Whole blood cell counts showed white blood cells (WBCs): 2.21 × 10^9^/L (normal: 4–10 × 10^9^/L), neutrophils: 0.08 × 10^9^/L (normal: 1.8–6.3 × 10^9^/L), red blood cells (RBCs): 1.37 × 10^9^/L (normal: 3.5–5.5 × 10^9^/L), hemoglobin (HGB): 4.0 g/dL (normal: 11–13 g/dL), mean corpuscular volume: 85 fL (normal: 82–100 fL), platelet count (PLT): 191 × 10^9^/L (normal: 100–300 × 10^9^/L), and reticular erythrocyte ratio: 2.21% (normal: 0.5–1.5%). Liver function tests demonstrated normal aminotransferases. Ferritin level was 313 ng/mL (normal: 13–150 ng/mL). 24-h urinary copper excretion was 30 μg. Serum copper level was low (56.3 μg/L, normal: 800–1290 μg/L). Serum zinc level was high (500 μg/dL, normal:75–150 μg/dL). Free copper level and 24-h urinary zinc excretion were not able to be tested. Renal function tests and urine analysis were unremarkable. Abdominal ultrasound revealed normal liver, spleen and gallbladder. There were no abnormal changes on brain Magnetic Resonance Imaging (MRI).Due to abnormal gait, studies of spinal MRI and cerebrospinal fluid were recommended, but rejected by her parents. In view of her severe anemia, 1 unit of erythrocyte suspension was transfused. We suspected that the anemia was possibly caused by hypocupremia secondary to zinc overtreatment. When zinc was withdrawn for 1 week, her hemoglobin level rose to 6.7 g/dL. She was then discharged and a normal diet was resumed. Low-dose zinc was recommended, but the girl’s parents refused the treatment though we told the possible flare of liver damage, even acute liver failure after the cessation of zinc treatment. The patient was regularly followed up with liver function tests, whole blood cell counts and indices of copper metabolism. At the age of 12 years and 2 months (4 months after discharge), her hemoglobin level normalized and normal gait was restored. The latest follow-up was in May 2018 (9 months after discharge), and her whole blood cell counts and liver function tests were all within the normal range. The patient’s hematological parameters, liver function tests, indices of copper and zinc metabolism are shown in Table 1.

**Table 1:** Laboratory findings of the present patient

	WBC (*10^9^/L)	Neutrophils (*10^9^/L)	RBC (*10^9^/L)	HGB (g/dL)	PLT (*10^9^/L)	ALT (IU/L)	AST (IU/L)	Serum copper (ug/L)	Serum Zinc (ug/dl)	24-h urine copper (ug)
7y^a^	6.9	1.35	4.66	13.7	183	324	294	NA	NA	360
7y1m	6.5	1.30	5.0	12.9	194	210	158	NA	NA	NA
7y6m	5.1	1.18	5.1	13.4	185	36	26	NA	NA	NA
8y	6.1	1.24	5.3	14.5	196	43	33	NA	NA	NA
11y 4m^b^	2.1	0.09	1.6	4.7	159	31	21	127.3	530	NA
11y 10m^c^	1.5	0.08	1.37	4.0	191	16	17	56.3	500	30
11y 10m^d^	2.2	0.11	2.27	6.7	251	20	18	NA	NA	NA
12y 2 m	4.54	2.27	5.28	11.7	225	13	22	NA	NA	NA

* : multiply by. a: age at initiation of zinc therapy. b: age at onset of anemia. c: age at withdrawal of zinc. d: 1 week after zinc withdrawal. *y* years, *m* months, *NA* not assessed

## Discussion and conclusions

Our patient originally presented with elevated liver aminotransferases, low serum ceruloplasmin, and increased 24-h urinary copper excretion. In addition, gene sequencing showed c.2122-1G > T and c.3044 T > C (p. L1015P) compound heterozygous mutations in ATP7B; thus, the diagnosis of WD was established. She was treated with a low-copper diet and zinc gluconate. Although we advised only avoid a few kinds of copper-rich food (shellfish, nuts, chocolate, mushrooms, and organ meats) [19], the family followed a very strict dietary copper restriction according to some propagations from the internet. Zinc is known to remove excess copper by inducing enterocyte metallothionein, which preferentially binds copper, prevents its absorption and enhances its excretion [20, 21]. The guidelines recommend 150 mg elemental zinc per day (75 mg/day for children < 50 kg in body weight) administered in three divided doses, 30 min before meals [3, 22], but administration of zinc is usually individualized according to clinical manifestation, biochemical indexes and 24-h urinary copper excretion [10].For patients on zinc maintenance therapy, 24-h urinary copper excretion, an indicator of blood free copper [3], should be monitored regularly and maintained between 30 and 75 μg; Besides, serum zinc levels and urinary excretion of zinc should be maintained above 125 μg/dL and 1.5-2 g/d respectively [19].Our patient was treated with high dose of zinc due to significantly elevated liver aminotransferases and 24-h urinary copper excretion. Abuduxikuer K and Wang JS conducted a retrospective study with an average 1.54-year follow-up revealed that a high dose of elemental zinc in Chinese children with pre-symptomatic WD had the same efficacy as the conventional dose and it took less time to normalize the liver function tests, with no influence on complete blood count parameters in the short-term [23]. It took 6 months for liver aminotransferases to normalize. The patient continued to receive high-dose zinc and was lost to follow-up for 3.3 years. At the age of 11.3 years, she was found to have severe anemia by the local heamatologist, without hemolysis or hemorrhage. She received erythrocyte transfusion, no cessation of zinc, which did not improve hypocupremia and anemia obviously, as Rau AR reported that anemia following penicillamine in a WD patient did not respond to treatment with hematinics [13]. The mechanism needs further study. When returned to our hospital for a follow-up, the patient was found to have low 24-h urinary copper excretion (30 μg) with low serum copper level (56.3 μg/L), and significantly elevated serum zinc level (500 μg/dL), suggesting hypocupremia due to high-dose zinc supplementation. Unfortunately, urinary zinc excretion test was not available at our institution. After the withdrawal of zinc with resumption of normal diet, hemoglobin levels were increased significantly. Low-dose zinc therapy was proposed to the patient, but parents refused despite of possibilities of liver damage or acute liver failure. Scheinberg IH et al. reported that eight patients died of hepatic decompensation or fulminant hepatitis after the withdrawal of decoppering therapy [24]. Moreover, the patient had been fed a strict low-copper diet all long which aggravated negative copper balance.

Eight WD patients with hypocupremia-associated anemia following zinc therapy have been reported in the literature [10–17]. Seven patients were adults. The only child among them was a 16-year-old boy who had anemia after initial penicillamine therapy for 2 year and anemia worsened after switching to high dose zinc for 9 months [13]. Five patients received over-dose zinc, while three received conventional dose. The anemia was severer in patients receiving high dose zinc treatment. Four individuals were treated with zinc monotherapy, one was treated with zinc plus trientine, and the remaining 3 patients took high-dose zinc with or after initial penicillamine administration. Whole blood counts in all these patients returned to normal after the appropriate treatment (1 patient after copper supplement, 1 patient after zinc dose adjustment, 4 patients after zinc withdrawal, and 2 patients managed with zinc withdrawal in combination with copper supplementation). Our patient presented with anemia and neutropenia due to hypocupremia following high-dose zinc monotherapy for 4 years and 4 months, and her hematological parameters recovered after zinc withdrawal for 4 months. Some physicians may suggest switching to D-penicillamine or trientine, as de-coppering drug, both of them can also lead to hypocupremia-related anemia, as previously reported [25, 26].The details regarding zinc therapy and anemia in these 9 patients are listed in Table 2.

**Table 2:** Characteristics of zinc-induced anemia in WD

Case source	Age	Zinc dose and course (Bold:high dose)	Chelator drug	HGB (g/dL)	Neutrophils (× 10^9^/L)	Bone marrow	Treatment	Time to normal blood counts
Linn et al[10]	43 y	**207** mg/d for 30y	No	Anemia	Neutropenia	Unknown	Zinc withdrawal	Unknown
Horvath et al[11]	40 y	**200** mg/d for 14y and **250** mg/d for 1y	Initial Penicillamine for 6 m	7.8	0.26	Ring sideroblasts	Zinc withdrawal	Unknown
Dzieżyc et al. [12]	18 y	72.7 mg/d for 6y	No	10	0.17	MDS excluded	Zinc withdrawal	2 months
Rau et al. [13]	16 y	**High dose** for 9 m	Initial Penicillamine for 2y	5.8	Neutropenia	MDS, ring sideroblasts	Zinc withdrawal and copper supplement	8 weeks
Cortese et al. [14]	51 y	**242** mg/d for 13y and **484** mg/d for 1y	No	6.5	0.25	MDS	Zinc dose reduction	Unknown
Foubert-Samier et al. [15]	43 y	142 mg/d for 25y	With Trientine 900 mg/d for 25y	10.6	0.94	Not performed	Zinc withdrawal	Unknown
Van den Hamer et al. [16]	56 y	**484** mg/d for 2y	Initial Penicillamine for 29y	Anemia	Neutropenia	Unknown	Copper supplement	Unknown
Mohamed et al. [17]	26y	80.7 mg/d for 13y	No	7.4	0.2	vacuolated myeloid and erythroid precursors	Zinc withdrawal and copper supplement	4 months
Present case	11 y 4 m	**240** mg/d for 4y4 m	No	40	0.08	MDS	Zinc withdrawal	4 months

Age: Age when presented with anemia, *y* years, *m* months, *MDS* Myelocytic dysplasia syndrome

There are multiple causes of hypocupremia, mainly including copper malabsorption induced by short bowel syndrome, gastrointestinal surgery, Menkes disease and excessive zinc exposure due to WD, acne, and zinc-containing dental fixatives [27]. The characteristics of hypocupremia-associated anemia in different etiologies are similar. It may present as significant neutropenia, all forms of anemia (normocytic, macrocytic or microcytic), or thrombocytopenia. Bone marrow biopsy shows vacuolated myeloid and erythroid precursors, myelocytic dysplasia syndrome (MDS) or ring sideroblasts [28]. The exact mechanism of hypocupremia-related anemia is unclear and may involve the followings: Copper is a cofactor in many enzymatic redox reactions and can stimulate the transformation of divalent iron to trivalent iron which participates in the hematopoietic process. Copper promotes release of iron from hepatocytes and iron absorption from enterocytes. Ceruloplasmin and transferrin are involved in iron transfer. Copper also accelerates the maturation and release of immature RBCs [29]. When copper is deficient, iron metabolism is disturbed, which affects the hematopoiesis process. Another copper dependent enzyme, cytochrome C oxidase, is also involved in the mechanism of anemia. Under low copper conditions, impaired cytochrome C oxidase affects the mobilization of stored iron, leading to iron deposition in tissues [30]. This may explain why the ferritin level was not low, different from iron-deficiency anemia. Because copper is a component of proteins which function in the structure and physiology of the central nervous system, hypocupremia can also cause demyelination of the central nervous system similar to vitamin B12 deficiency. Typical features include abnormal gait, paresthesias, myasthenia, hypertonia, or hyperreflexia. The MRI of brain is usually normal, while T2-hyperintense signal can be seen in the dorsal columns of the spinal cord [31]. Our patient had a gait disorder, without other clinical symptoms of ataxia, myelopathy, and peripheral neuropathy. There were no abnormal changes on brain MRI. The reason for abnormal gait could not be ascertained because further studies of spinal cord MRI, and cerebrospinal fluid were refused by parents. Usually it may take longer time to develop copper deficiency in WD, but due to our patient’s very strict diet control, higher zinc dose, and her relative low body weight, it took a shorter time to remove the excess copper accumulation in her body. The patient had not been back to us for years which prevented our clinicians identifying possible adverse effects promptly. Her hypocupremia-associated anemia was not diagnosed until she returned to our liver specialist.

In conclusion, we report the first child patient with hypocupremia-associated anemia which developed following high-dose zinc monotherapy for Wilson’s disease. Anemia during zinc therapy should prompt clinicians to exclude zinc over-treatment. Furthermore, this case demonstrates the importance of regular follow-up of whole blood cell counts, indices of copper and zinc metabolism, in the long-term management of WD [32], especially in whom high-dose zinc is administrated.

## Data Availability

All data generated or analyzed during this study are included in this published article.

## Abbreviations

HGB: Hemoglobin
MDS: Myelocytic dysplasia syndrome
MRI: Magnetic Resonance Imaging
PLT: Platelet count
RBC: Red blood cell
WBC: White blood cell
WD: Wilson’s Disease
